# Supplementary material for: Exotic and native plants play equally important roles in supporting and structuring plant-hummingbird networks within urban green spaces
Source: PeerJ. 2024 Feb 21;12:e16996. doi: 10.7717/peerj.16996 (PMC10893870; doi:10.7717/peerj.16996)
Supplement: Supplemental Information 2 — The plots illustrate the monthly fluctuations in recorded flower (A and B) and hummingbird (C) abundance observed over a year. Each data point represents the count of flowers and hummingbirds per species recorded during specific months throughout the year. The line in the plot depicts a smoothed trend, providing an overview of the trend in flower and hummingbird abundance over the entire year. [file peerj-12-16996-s002.docx]

Supplemental material for: **Exotic and native plants play equally important roles in supporting and structuring plant-hummingbird networks within urban green spaces**

Figure 1SA


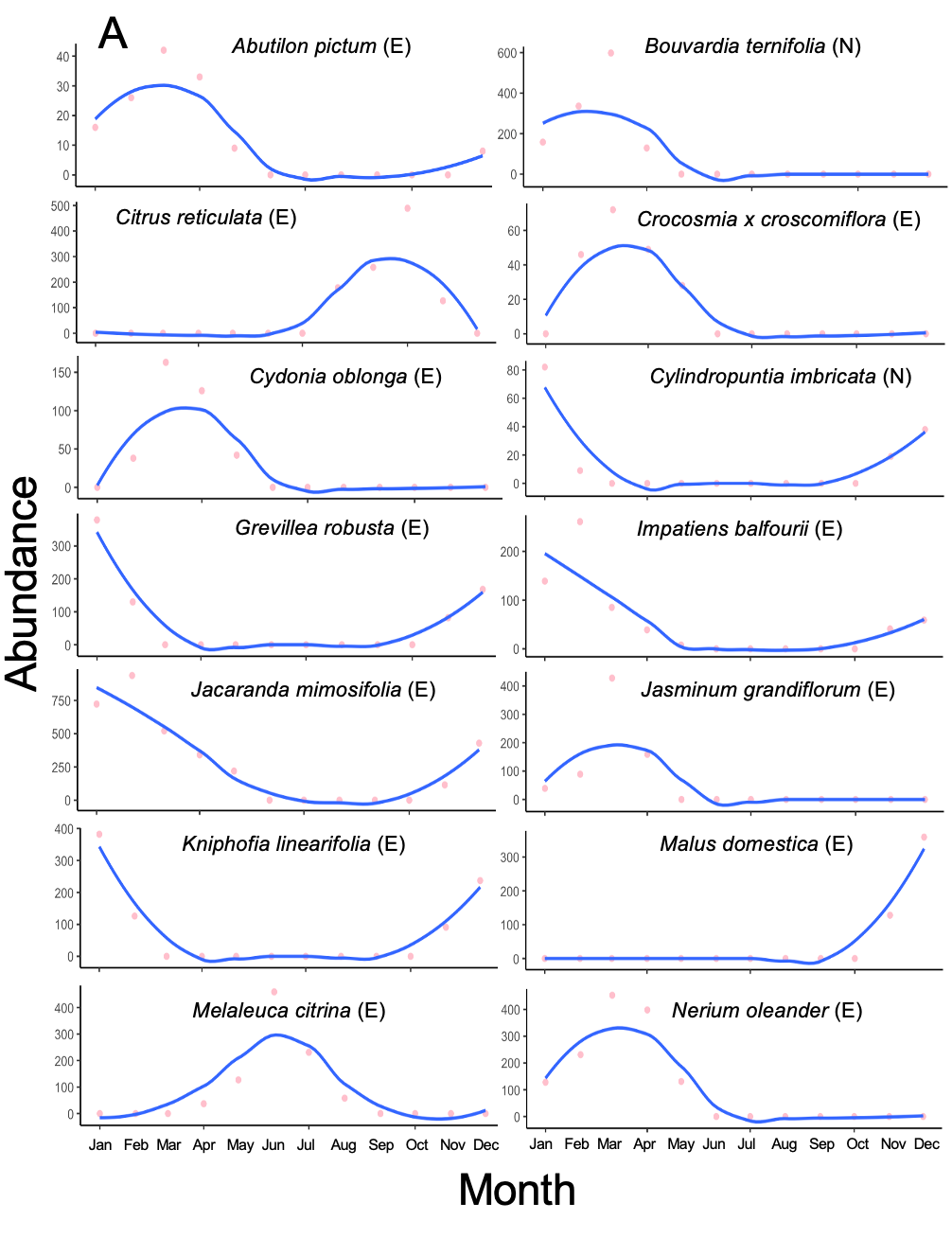


Figure 1 SB


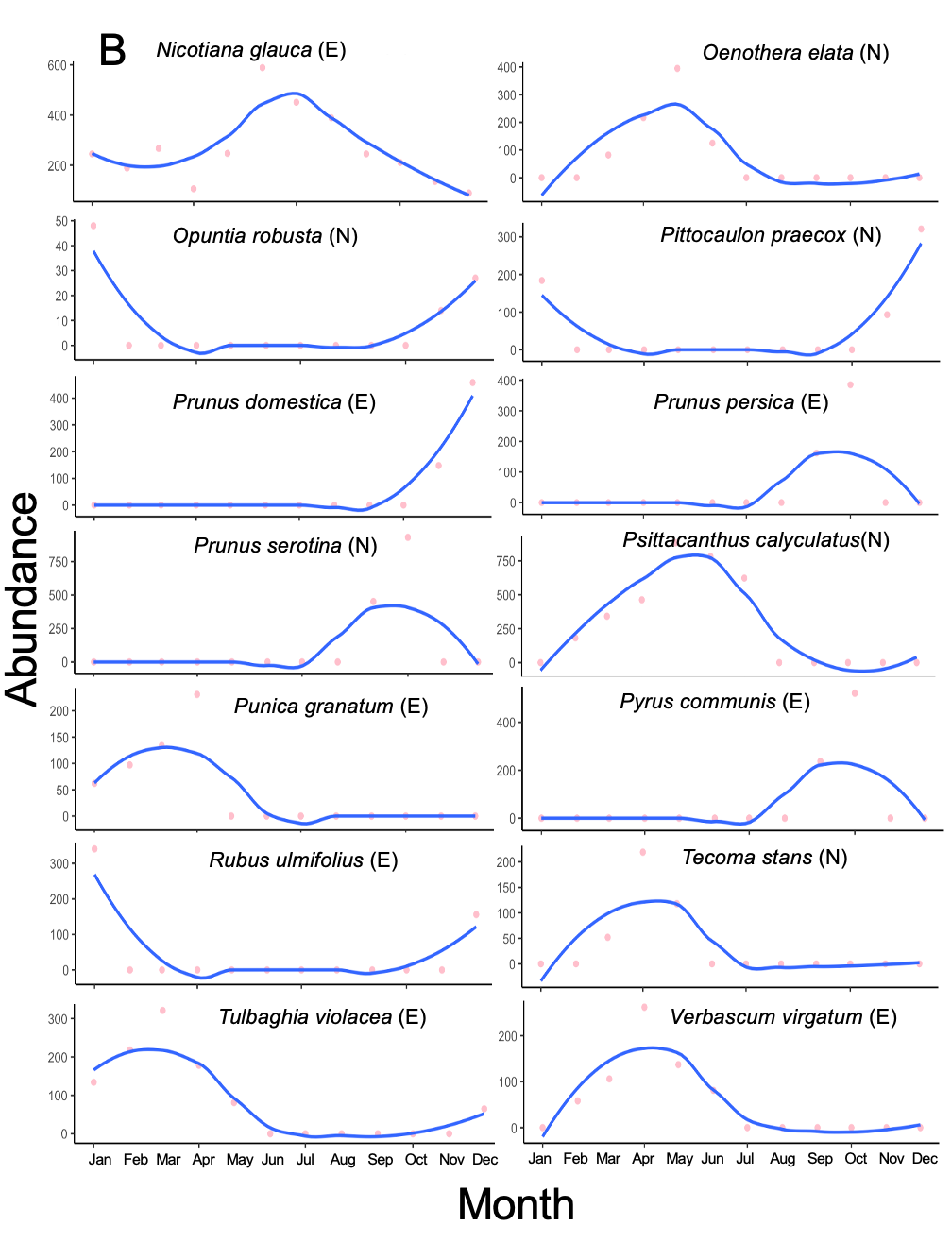


Figure 1SC


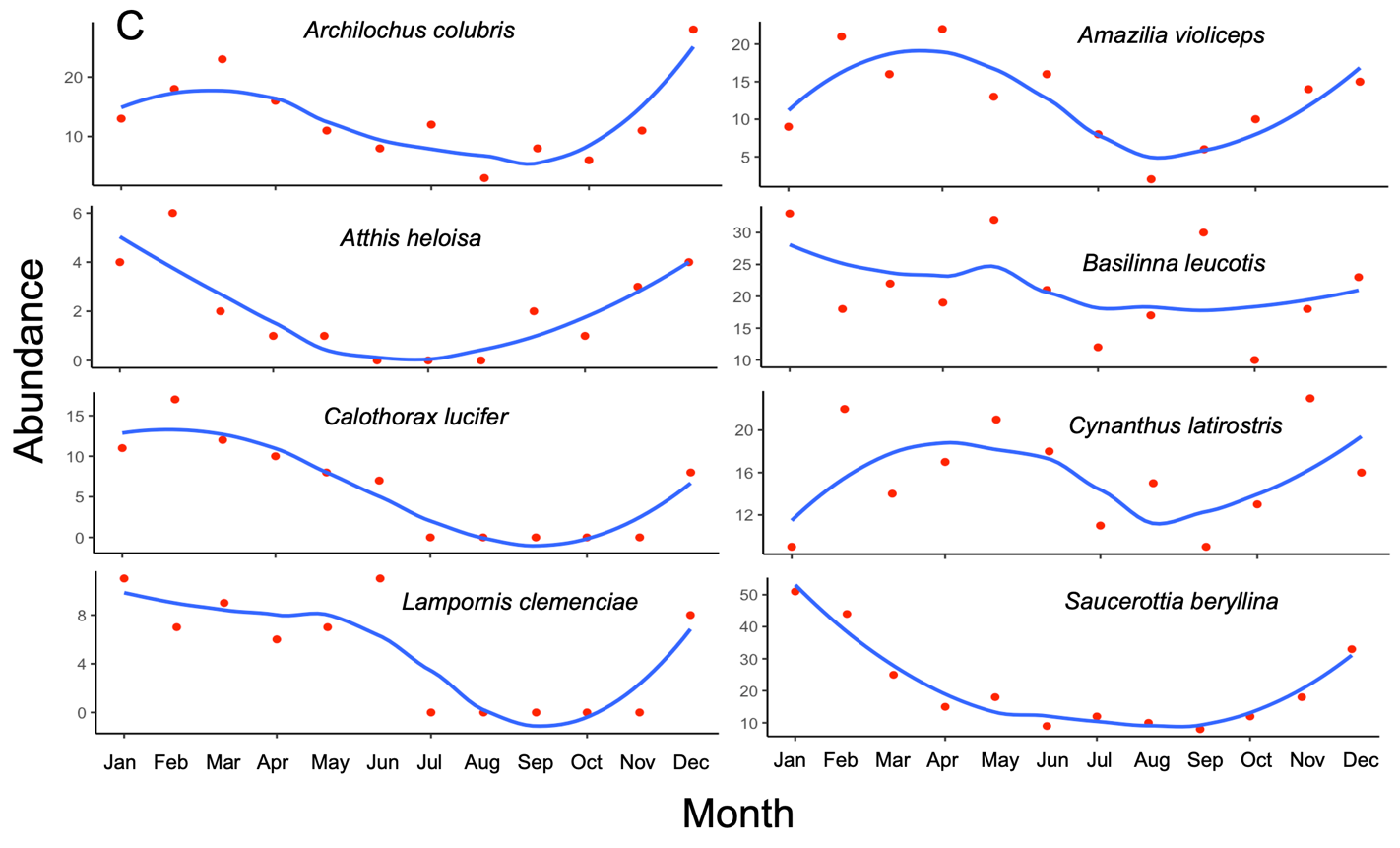


**Figure S1: Monthly variations in flowers and hummingbirds recorded at the TBG (Tizatlán Botanical Garden, Tlaxcala, México).** The plots illustrate the monthly fluctuations in recorded flower (A and B) and hummingbird (C) abundance observed over a year. Each data point represents the count of flowers and hummingbirds per species recorded during specific months throughout the year. The line in the plot depicts a smoothed trend, providing an overview of the trend in flower and hummingbird abundance over the entire year.
